# Synthesis and characterisation of alkyd resins with glutamic acid-based monomers†

**DOI:** 10.1039/c8ra00060c

**Published:** 2018-02-21

**Authors:** Joris Hulsbosch, Laurens Claes, Dries Jonckheere, Dirk Mestach, Dirk E. De Vos

**Affiliations:** KU Leuven, Department of Microbial and Molecular Systems, Centre for Surface Chemistry and Catalysis Celestijnenlaan 200F, Box 2461 3001 Leuven Belgium dirk.devos@kuleuven.be; Allnex Netherlands B.V. Synthesebaan 1 (4612 RB), P. O. Box 79 4600 AB Bergen op Zoom The Netherlands

## Abstract

Alkyd resins are versatile polymers which have applications in inks and various coatings like decorative paints. They are mainly composed of fatty acids, polyols and aromatic diacids. In this work, glutamic acid as well as *N*-acylated and *N*-alkylated derivatives there of were evaluated as bio-based substitutes for these aromatic diacid monomers in the synthesis of alkyd resins. The resins were characterised in terms of structure, molecular weight, viscosity, oxidative thermal stability and colour. *N*-Palmitoylglutamic acid dimethyl ester can be successfully incorporated when the polycondensation is performed in two steps. In this approach, the bio-based diacid monomer is only supplied in the second step, because the removal of water in the first step is essential to avoid hydrolysis of the monomer amide bond and the subsequent formation of pyroglutamate groups. The molecular weight, viscosity and oxidative thermal stability are lower than for conventional alkyd resins. The mechanism of the discolouration of alkyd resins during polymerisation is mediated by free radical species, which were generated easily in the presence of free amino groups and/or unsaturated fatty acids. Light-coloured resins could be obtained by using saturated fatty acids or radical scavengers during polymerisation.

## Abstract 2

Novel alkyd polyester resins for the paint industry using biobased glutamic acid instead of petrochemicals.

## Graphical abstract

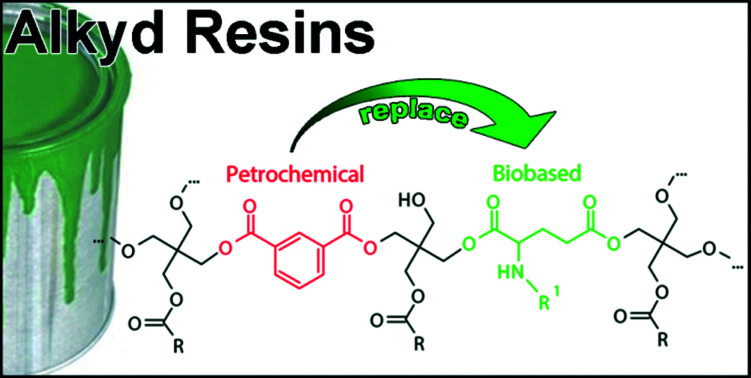

## Introduction

Alkyd resins nowadays have major applications as binders in the manufacture of paints and coatings. These polymers consist of a polyester backbone prepared from polyols and acid anhydrides or diacids such as maleic anhydride, phthalic anhydride and isophthalic acid.^1^ The residual backbone hydroxyl groups are esterified by long-chain unsaturated fatty acids. Upon contact with air during drying, cross-linking of the polymer chains will be induced by oxidation of the unsaturated bonds, resulting in a polymer film that provides the structural component of the paint.^1,2^

Although the interest in alkyd resins had diminished since the discovery of acrylic resins as more efficient binders,^1^ they have regained a more prominent position as a result of today's trend towards the development of more sustainable industrial processes and products. Alkyd resins are typically composed of up to 90% bio-based constituents,^3^ in particular fatty acids and glycerol or pentaerythritol, which are easily obtained from biofuels production and agro-industrial waste.^4,5^ However, the diacid building blocks are still derived from petrochemical resources. A number of bio-based alternatives, such as succinic acid,^3^ ferulic acid^6^ and 2,5-furandicarboxylic acid^7,8^ have been proposed for the synthesis of polyesters. Succinic acid can be produced by fermentation from glucose and this C4-diacid can replace 25–100% of the aromatic diacid content in alkyd resins. In general, these alkyd resins show a higher flexibility, lower glass transition temperature and less yellowing. However, the use of aliphatic diacids like succinic acid can cause problems concerning premature gelling of the resin which renders it unusable.^3^ Ferulic acid can be produced from lignin and has been used in the synthesis of polyferulic acid by condensation polymerisation.^6^ This polyester has thermal properties comparable to those of polyethylene terephthalate (PET). 2,5-Furandicarboxylic acid can also be obtained from carbohydrates and has been used commercially to produce poly(ethylene 2,5-furandicarboxylate), a PET analogue.^7,8^ Therefore both ferulic acid and 2,5-furandicarboxylic acid are also expected to be useful co-monomers in the preparation of alkyd resins.

Glutamic acid can be proposed as another bio-based diacid to replace conventional aromatic diacids in polyester synthesis. This non-essential amino acid is abundantly available on a million ton scale *via* fermentation.^9,10^ Moreover, as glutamic acid is a major constituent of plant proteins, agro-industrial waste streams can be considered as an additional resource.^11^ In this work the feasibility of incorporating glutamic acid as a monomer in alkyd resin synthesis is studied. The resulting resins are compared to a conventional alkyd resin that exclusively contains non-renewable diacids.

## Results and discussion

### Synthesis of alkyd resins

**Scheme 1:**
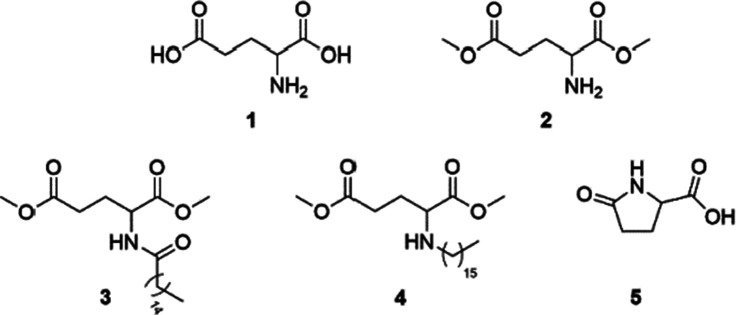
Glutamic acid (1) and its derivatives: glutamic acid dimethyl ester (2), *N*-palmitoylglutamic acid dimethyl ester (3), *N*-hexadecylglutamic acid dimethyl ester (4) and pyroglutamic acid (5).

Several alkyd resins were prepared by treating a mixture of fatty acid(s), a polyol and various diacids at high temperature. Isophthalic acid, which is typically applied in the industrial production of alkyd resins, was in this study partially replaced by a bio-based, non-aromatic diacid. The composition and the synthesis conditions are summarised in Table 1. First, a benchmark material was synthesised by heating a mixture of soy bean oil fatty acids (72 wt%), pentaerythritol (17 wt%), isophthalic acid (6 wt%) and 1,2,3,6-tetrahydrophthalic acid (5 wt%) at 210 °C for 18 h (resin A). Mesitylene (3 wt%) was added to the reaction medium to enable the azeotropic removal of water and to avoid formation of tarry residus in the set-up, but was evaporated afterwards. A next alkyd resin was prepared by substitution of 1,2,3,6-tetrahydrophthalic acid with glutamic acid (resin B). This amino acid is however known to be unstable at temperatures > 120 °C, and is expected to degrade rapidly by intramolecular condensation to pyroglutamic acid under typical reaction conditions.^12^ Moreover, free amino groups on the polymer chain may react with radical species generated by oxidation of the unsaturated fatty acid side chains, eventually resulting in discolouration of the resin. Although dark-coloured resins can still be applied in printing inks, this phenomenon is especially undesired regarding the potential application of these bio-based alkyd resins in the formulation of white or light-coloured decorative paints. *N*-Acylated and *N*-alkylated derivatives of glutamic acid dimethyl ester (Scheme 1, 2–5) are expected to be more stable towards cyclisation and were therefore evaluated as co-monomers in alkyd synthesis (resins C–F). The fate of *N*-acylated pyroglutamic acid methyl ester under typical conditions was also studied (resin G). In general, modification of the amino group in (pyro)glutamic acid by long-chain alkyl or acyl fragments will increase the hydrophobicity of the amino acid and hence improve the miscibility with other apolar compounds in the reaction medium. The influence of a radical scavenger such as butylated hydroxytoluene on the extent of resin discolouration was also evaluated (resin F). Finally, in order to simplify product characterisation, several alkyd resins were prepared from palmitic acid and oleic acid instead of soy bean oil fatty acids, which is an impure mixture of both unsaturated and saturated fatty acids (resins H and I).

**Table 1:** Synthesis and characterisation of glutamic acid-based alkyd resins

Resin	Synthesis^a^			Characterisation		
	Fatty acid source	Diacid or derivative (+additive)	Conditions	Acid value^b^, (mg KOH per g resin)	Mol. weight^c^, (g mol^−1^)	Viscosity^d^, (Pa s)
A	Soy bean oil fatty acids	1,2,3,6-Tetrahydrophthalic acid^e^	18 h at 210 °C	10.7	2573	1.2
B	Soy bean oil fatty acids	Glutamic acid^e^	18 h at 210 °C	5.7	1734	1.0
C	Soy bean oil fatty acids	*N*-Palmitoylglutamic acid dimethyl ester^e^	18 h at 210 °C	2.3	1762	0.34
D	Soy bean oil fatty acids	*N*-Palmitoylglutamic acid dimethyl ester^f^	(1) 6 h at 210 °C, (2) 18 h at 160 °C	5.4	4070	2.4
E	Soy bean oil fatty acids	*N*-Hexadecylglutamic acid dimethyl ester^f^	(1) 6 h at 210 °C, (2) 18 h at 160 °C	3.7	1923	0.043
F	Soy bean oil fatty acids	*N*-Palmitoylglutamic acid dimethyl ester + butylated hydroxytoluene^e^ (1 equiv.)	18 h at 210 °C	3.2	n.d.^g^	n.d.^g^
G	Soy bean oil fatty acids	*N*-Palmitoylpyroglutamic acid methyl ester^e^	18 h at 210 °C	7.4	1660	0.092
H	Palmitic acid	*N*-Palmitoylglutamic acid dimethyl ester^f^	(1) 6 h at 210 °C, (2) 18 h at 160 °C	5.1	1630	Solid
I	Oleic acid	*N*-Palmitoylglutamic acid dimethyl ester^f^	(1) 6 h at 210 °C, (2) 18 h at 160 °C	6.3	n.d.^g^	Solid

a General composition of the synthesis mixture: fatty acid(s) (72 wt%), pentaerythritol (17 wt%), isophthalic acid (5 wt%), diacid or derivative (6 wt%) and mesitylene (3 wt%).

b Determined by acid–base titration.

c Measured by gel permeation chromatography.

d Measured by rheometry at 23 °C.

e The diacid was added at the onset of polymerisation.

f The diacid was added after 6 h.

g n.d. = not determined.

The polymerisation was performed either in one step at 210 °C (resins B, C, F and G) or in two steps (resins D, E, H and I). In the latter approach, the polycondensation of the fatty acids, pentaerythritol and isophthalic acid proceeded first at 210 °C for 6 h, and after addition of the bio-based diacid co-monomer the polymerisation was continued at 160 °C for 18 h. The diacid co-monomer will be incorporated into the polyester backbone by transesterification. The aim is to remove water from the reaction medium in the first step, in order to avoid the hydrolysis of the fatty amide in glutamic acid dimethyl ester derivatives (3) and the subsequent formation of pyroglutamate rings.

### Characterisation of alkyd resins

The resins were characterised in terms of structure, molecular weight, viscosity and oxidative thermal stability (Table 1).

### Degree of polymerisation (DP)

The DP of the resins was expressed by the acid value, which is an industrial measure for the conversion and can be determined by titration with an ethanolic solution of potassium hydroxide (KOH). The acid value of the glutamic acid-based alkyd resins was below 10 mg KOH per g resin (Table 1), which is in accordance with the industrial standards.^1^ These values reveal that the amount of residual free carboxylic acid groups in the resins is low, hence that the majority of the diacid monomers and fatty acids have been involved in the polycondensation.

### Molecular structure

**Fig. 1:**
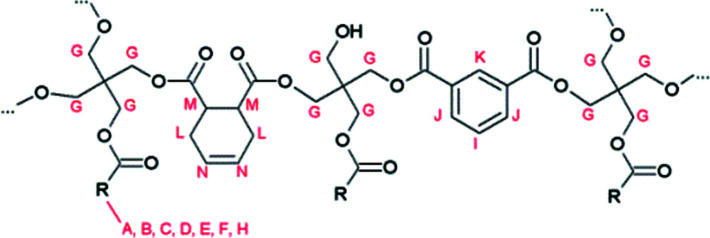
Proposed structure of resin A, with R being the alkyl chain of a fatty acid. Characteristic signals in the ^1^H NMR spectrum are indicated.

The molecular structure of the resins was determined by nuclear magnetic resonance (NMR) spectroscopy. The ^1^H NMR spectrum of reference resin A, which contains 1,2,3,6-tetrahydrophthalic acid as a non-aromatic diacid co-monomer, shows characteristic signals of the (unsaturated) fatty acid side chains, pentaerythritol and isophthalic acid (Fig. 1 and 3).

**Fig. 2:**
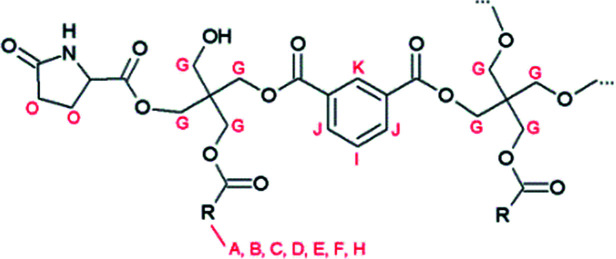
Proposed structure of resin B, with R being the alkyl chain of a fatty acid. Characteristic signals in the ^1^H-NMR spectrum are indicated.

**Fig. 3:**
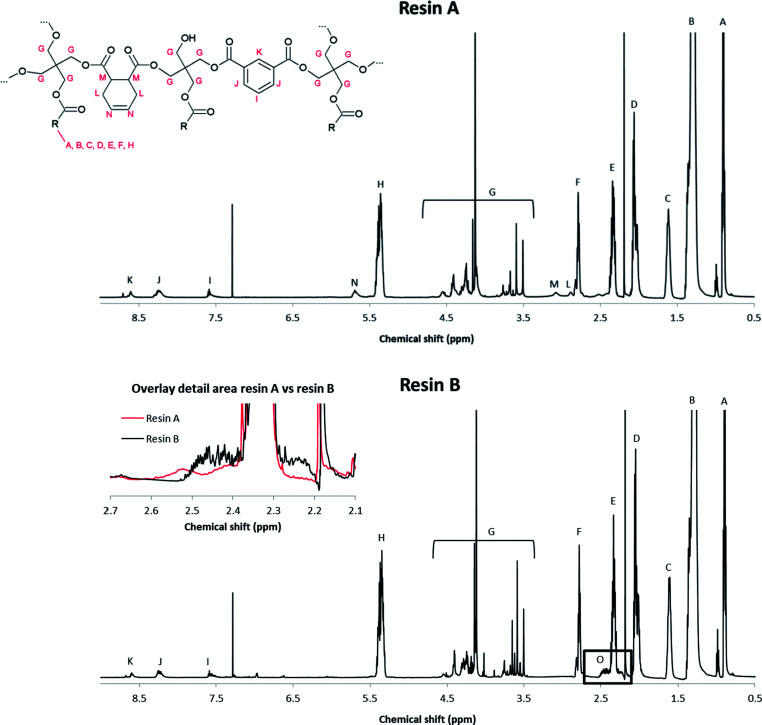
600 MHz ^1^H NMR spectra of resins A and B.

These signals were also observed in the ^1^H NMR spectrum of resin B, which was prepared with glutamic acid (Fig. 3).

In addition, a new multiplet signal at 2.4–2.5 ppm appeared in the spectrum of this resin, which can be unequivocally ascribed to the presence of pyroglutamate groups in the sample. Remarkably, although pyroglutamic acid, as a lactam derivative of glutamic acid, is insoluble in deuterated chloroform, resin B dissolved completely in this apolar medium. From this observation it can be deduced that pyroglutamates were incorporated in the polymer matrix as monofunctional chain stoppers (Fig. 2), rather than being present as a free monomer.

Chain stoppers are often incorporated in commercial alkyd resins to control the molecular weight of the resin.^1^ In this way, the physical and chemical properties of the resin, such as viscosity, drying time and flexibility of the polymer film can be adjusted. Pyroglutamic acid can thus be considered as a bio-based alternative to non-renewable chain stoppers such as benzoic acid or 4-*tert*-butylbenzoic acid.^13^

**Fig. 4:**
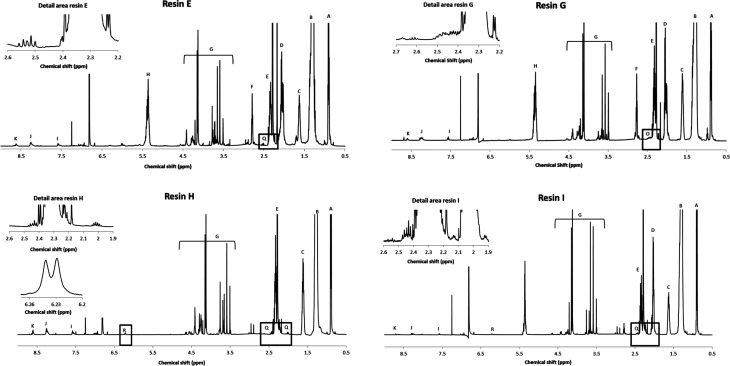
600 MHz ^1^H NMR spectra of resins E, G, H and I.

**Fig. 5:**
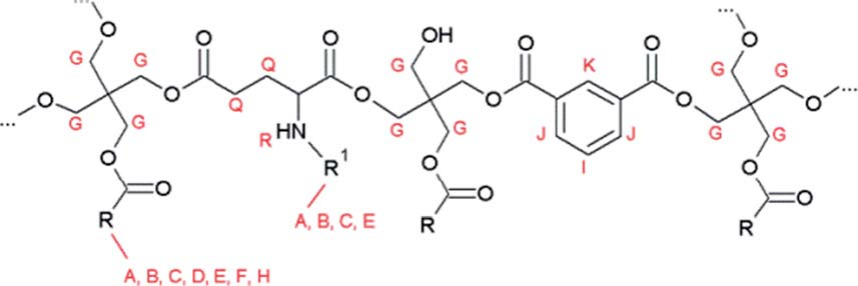
Proposed structure of resin E, H and I with R being the alkyl chain of a fatty acid and R^1^ represents a hexadecyl or hexadecanoyl moiety.

The cyclisation of glutamic acid to pyroglutamic acid cannot be prevented under typical reaction conditions (*e.g.*, 210 °C) and therefore *N*-acylated and *N*-alkylated derivatives of glutamic acid dimethyl ester were evaluated as diacid co-monomers in combination with isophthalic acid. The ^1^H NMR spectrum of resin C, where *N*-palmitoylglutamic acid dimethyl ester was added at the onset of the polymerisation, demonstrates that the formation of pyroglutamic acid can be prevented by this approach: the multiplet of pyroglutamate, at 2.4–2.5 ppm, is not observed in the spectrum (Fig. S9†). There is however no evidence for the incorporation of this diacid building block into the polymer chain due to overlapping signals in the spectrum. Moreover, an additional complication could be the hydrolytic degradation of *N*-palmitoylglutamic acid dimethyl ester under these harsh conditions. Similar ^1^H NMR spectra, without pyroglutamate peaks, were obtained for resins D and F (Fig. S10 and S11†), where *N*-palmitoylglutamic acid dimethyl ester was added in the second phase of the polymerisation, or where a radical scavenger was supplied to the reaction medium, respectively. Pyroglutamate groups were also absent in resin E, which was prepared by the addition of *N*-hexadecylglutamic acid dimethyl ester as a co-monomer in the second phase. Moreover, the incorporation of this *N*-alkylated glutamic acid derivative into the polymer matrix in a linear, non-cyclic way, was supported by a characteristic signal at 2.5 ppm in the corresponding ^1^H NMR spectrum (Fig. 4 and 5).

Analysis of resin G revealed the incorporation of pyroglutamic acid in the polymer matrix, in a similar, chain-stopping manner as in resin B (Fig. 2), suggesting that hydrolysis of *N*-palmitoylpyroglutamic acid methyl ester occurs when it is added at the onset of the polymerisation. Resins H and I were synthesised using palmitic acid and oleic acid respectively, and *N*-palmitoylglutamic acid dimethyl ester was added after an initial phase of polymerisation, analogously as for resin D. The corresponding ^1^H NMR spectra clearly show that the *N*-acylated derivative of glutamic acid was incorporated into the polymer chain (Fig. 4). The multiplet signals at 2.0 ppm and 2.4 ppm as well as the doublet signal at 6.24 ppm are characteristic for the *N*-palmitoylglutamic acid moiety. The presence of residual free diacid monomers in the resin matrix was ruled out after evaluation of the thermal stability of the precursor. To that end, *N*-palmitoylglutamic acid dimethyl ester was dissolved in mesitylene and heated under reflux conditions (164 °C) for 6 h. Since this compound was degraded completely within 4 h, this glutamic acid derivative can only be stable towards cyclisation by virtue of its incorporation into the polymer. The molecular structure is therefore expected to be similar to the one presented in Fig. 5.

### Molecular weight and viscosity

The molecular weight and the viscosity of the resins were determined by gel permeation chromatography (GPC) and rheometry respectively (Table 1). For commercial alkyd resins these parameters are in the range of 7000–24 000 g mol^−1^ and 1–7.6 Pa s at 23 °C.^14^ The molecular weight of resin A was about 2570 g mol^−1^, which is relatively low compared to the industrial standard. Most glutamic acid-based resins even have a lower molecular weight, ranging from 1630 g mol^−1^ to 1920 g mol^−1^. These results can be explained in terms of temperature: the synthesis of alkyd resins was performed at <210 °C in this study, whereas temperatures up to 270 °C are typically applied in industry.^1^ Nevertheless, these measurements support the beneficial effect of the two-step procedure, because resin D has a much higher molecular weight (4070 g mol^−1^) compared to resin C. The viscosity characteristics are in accordance with the molecular weight values; in general, resins with a higher molecular weight display a higher viscosity. The viscosity could not be measured for resins H and I: because of their saturated chains, they are solids at 23 °C.

### Oxidative thermal stability

**Fig. 6:**
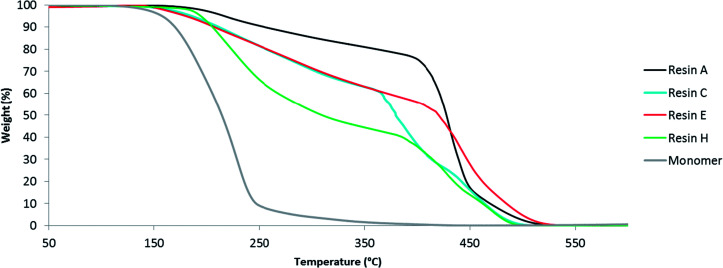
Thermogravimetric analysis of several alkyd resins and *N*-palmitoylglutamic acid dimethyl ester monomer.

During industrial production of alkyds, there might be some oxygen present. In order to determine the oxidative thermal stability of the resin in these conditions, they were analysed by thermogravimetric analysis (TGA) under a flow of oxygen. The benchmark resin A and most glutamic acid-based resins started to degrade slowly above 200 °C (Fig. 6, S12 and S13†). The degradation was accelerated at 400 °C and the resins were decomposed completely at 550 °C. Resin H provides an exception, because the thermal degradation was already pronounced at 200 °C. This behavior can be explained by the higher fraction of *N*-palmitoylglutamic acid that was incorporated into resin H. Moreover, the extent of cross-linking between the polymer chains is expected to be reduced. Comparison of resins C and E shows that resins containing *N*-palmitoylglutamic acid dimethyl ester are slightly more unstable than those containing *N*-hexadecylglutamic acid dimethyl ester. In general, the thermal stability of the resins was reduced by the incorporation of glutamic acid or derivatives thereof, because these bio-based diacid co-monomers degrade at much lower temperature than conventional diacids such as isophthalic acid. For instance, the degradation of *N*-palmitoylglutamic acid dimethyl ester was nearly complete at 250 °C (Fig. 6). Nevertheless, the higher thermal stability of the resins compared to the free diacid monomers provides additional evidence for the incorporation of glutamic acid derivatives into the resin matrix. These resins are mainly intended for applications in paint and ink formulation and therefore the lower thermal stability is not expected to be an issue because typical industrial processes proceed far below the degradation temperature.

### Radical-mediated discolouration mechanism

**Fig. 7:**
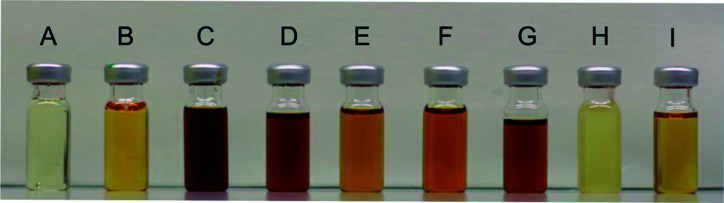
Comparison of resins A–I in terms of colour.

The discolouration of the alkyd resins was evaluated qualitatively (Fig. 7). Remarkably, resin H, without any double bonds in the side chain, was nearly uncoloured compared to resin I, which is based on oleic acid and assumes a darker hue after polymerisation. The discolouration was less pronounced in the presence of a radical scavenger (resin F) or when the synthesis was performed with a saturated fatty acid (resin H). These observations support a radical-mediated discolouration mechanism, because the generation of free radicals was expected to be strongly reduced, either when the fatty chains are saturated (as in H), or when a radical trap is added (as in F).

**Scheme 2:**
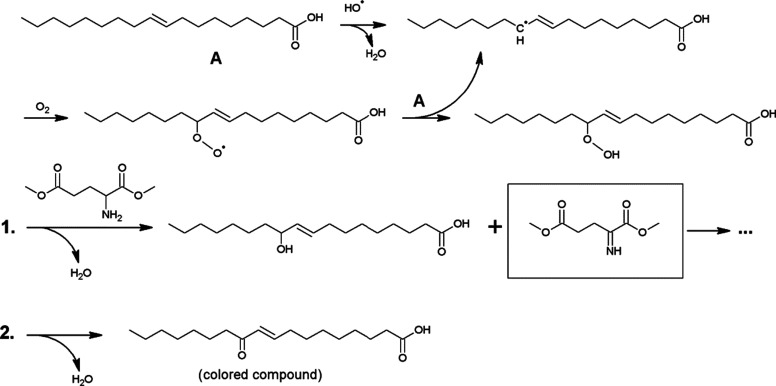
Proposed radical pathways for the initiation of resin discolouration: allylic C–H abstraction in the unsaturated fatty acid by reactive oxygen species, followed by the generation of an allylic alkyl hydroperoxide intermediate, which reacts with glutamic acid dimethyl ester to generate an imine and a fatty acid-derived allylic alcohol (route 1) or undergoes dehydration to an α,β-unsaturated ketone (route 2).

A hypothetical mechanism is proposed in Scheme 2. Free radicals can be generated during the synthesis of alkyd resins at high temperature in the presence of traces of gaseous oxygen. Reactive oxygen species such as HO˙ can easily abstract an allylic hydrogen atom from an unsaturated fatty acid side chain and the subsequent formation of fatty acid alkyl hydroperoxides proceeds very fast even at low oxygen concentrations. The primary amine in glutamic acid can be dehydrogenated by the alkyl hydroperoxide, with formation of an imine (Scheme 2, route 1).^15^ The latter is not stable during polymerisation and will be converted to *N*-oxides, aminals, tetrahydropyridines and other products that can induce colour formation.^16^ Both the modification of the primary amine in glutamic acid and the use of saturated fatty acids are thus effective measures to prevent discolouration of the resin. Furthermore, fatty acid alkyl hydroperoxides can be dehydrated to α,β-unsaturated fatty acid ketones (Scheme 2, route 2), which are responsible for conventional yellowing of alkyd resins.^17^ This process is even more pronounced in the presence of poly-unsaturated fatty acids.^18^ For instance, resins B–G which were prepared from a soy bean oil fatty acid mix containing *e.g.* linoleic acid, were much darker compared to the analogous resin I which was prepared from oleic acid.

## Conclusion

A number of alkyd resins were synthesised using glutamic acid and derivatives thereof as bio-based diacid monomers, in combination with conventional non-renewable aromatic diacids. In all cases, the degree of polymerisation corresponds to the industrial standards. The lactamisation of glutamic acid to pyroglutamic acid occurs as a competing side reaction at high temperature and should be avoided, because this compound is incorporated as a chain stopper, which is unfavourable in terms of molecular weight. To that end, the primary amine in glutamic acid can be modified by acylation or alkylation. Moreover, the hydrolytic degradation of *N*-acylated derivatives of glutamic acid can be prevented by performing the polymerisation in two steps, where a polyester backbone is formed in the first step and the glutamic acid-based monomers are incorporated by transesterification in the second step.

The molecular weight, viscosity and thermal stability of the bio-based resins were relatively low in comparison with commercial alkyd resins. Moreover, discolouration of the resins occurs to some extent, because the amine moiety of glutamic acid and the unsaturated fatty acids are involved in free radical reactions. Nevertheless, to the best of our knowledge, glutamic acid has been used for the first time as a difunctional polymer building block for the synthesis of bio-based, and even colourless alkyd resins.

## Experimental

### Synthesis of *N*-acylated and *N*-alkylated derivatives of glutamic acid

All reactants were purchased and used without further purification. *N*-Palmitoylglutamic acid dimethyl ester was synthesised by *N*-acylation of the amino acid according to the Schotten–Baumann method.^19,20^ To that end, glutamic acid dimethyl ester hydrochloride (10 g) was dissolved in dry methanol (100 ml). After addition of sodium methoxide base (1 equiv., 2.56 g), the mixture was chilled, filtered and carefully concentrated under vacuum. Next, glutamic acid dimethyl ester free base (8 g) was dissolved in *N*,*N*-dimethylformamide (DMF; 50 ml), followed by the addition of pyridine (1.3 equiv., 4.7 g) and a solution of palmitoyl chloride (1 equiv., 12.5 g) in DMF (20 ml). The mixture was stirred at room temperature for 1 h and chilled at 4 °C overnight. The precipitate was filtered and the desired product was recrystallised from hexane (43% isolated yield). An analogous procedure was applied for the synthesis of *N*-palmitoylpyroglutamic acid methyl ester (42% isolated yield).

The *N*-alkylation of glutamic acid was performed according to the procedure of Yu and co-workers.^21^ To that end, glutamic acid dimethyl ester (10 g) was dissolved in DMF (100 ml) and the reaction vessel was placed in an ice bath at 0 °C and kept under nitrogen atmosphere. Sodium hydride (1.3 equiv., 60% dispersion in mineral oil, 3 g) was added slowly, and after 30 min also hexadecyl bromide (1.2 equiv., 17.4 g). The mixture was stirred at room temperature overnight. Afterwards, the mixture was carefully poured into a saturated aqueous solution of ammonium chloride (100 ml), diluted with the same volume of water and extracted twice with ethyl acetate (200 ml). The combined organic phases were concentrated under vacuum and the product was obtained in 50% yield. ^1^H NMR and GC-MS data are provided in the ESI.†

### Synthesis of alkyd resins

Alkyd resins were synthesised in a three-necked round-bottom flask equipped with a Dean–Stark apparatus for water removal, a reflux condenser, a magnetic stirring bar, a heating mantle and a temperature controller. The procedure is illustrated here for the synthesis of resin H. The reaction mixture was composed of palmitic acid (72 wt%, 31.5 g), pentaerythritol (17 wt%, 7.6 g), isophthalic acid (5 wt%, 2.25 g), *N*-palmitoylglutamic acid dimethyl ester (6 wt%, 2.7 g) and mesitylene (3 wt%, 1.35 g); the total weight was approximately 45 g. All reactants except for the *N*-acylated derivative of glutamic acid were loaded into the flask and the recipient was heated at 210 °C for 6 h. Afterwards, *N*-palmitoylglutamic acid dimethyl ester was added and the polymerisation was continued at 160 °C for 18 h. Finally, mesitylene and other volatile compounds were removed by drying in a vacuum oven at 80 °C overnight.

### Characterisation of alkyd resins

#### Degree of polymerisation (DP)

The acid value of the resin, which is an industrial measure for the DP, was determined by acid–base titration. To that end, a sample of the resin was dissolved in ethanol (*e.g.* 5 g in 50 ml). After addition of phenolphthalein as a colour indicator the solution was titrated with a 0.2 M solution of potassium hydroxide (KOH) in ethanol. The acid value was calculated according to the formula:
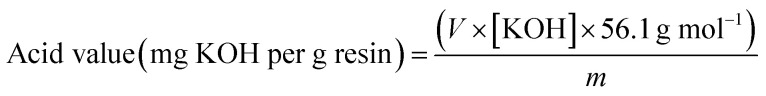
with: *V* = volume of the KOH solution added until neutralisation (ml); [KOH] = concentration of the KOH solution (M); *m* = weight of the resin sample (g).

#### Nuclear magnetic resonance (NMR) spectroscopy

The molecular structure of the resins was determined from ^1^H NMR measurements, which were performed on a Bruker Avance 600 MHz spectrometer. Samples were dissolved in deuterated chloroform (*e.g.* 70 mg in 0.7 ml). ACD labs software was used for data analysis.

#### Gel permeation chromatography (GPC)

The molecular weight of the resins was determined by using a Shimadzu 10A GPC system equipped with a PLgel 5 μm mixed-D type column, a differential refractometer and a UV-VIS spectrophotometer. Data analysis was performed using Empower GPC software.

#### Rheometry

The viscosity of the resins was measured on a Rheolab MC1 Physica rotational rheometer at 23 °C.

#### Thermogravimetric analysis (TGA)

The oxidative thermal stability of the resins was determined by using a TA Instruments Q500 thermogravimetric analyser. Samples were dried first under vacuum (10 mbar) at 80 °C for 18 h, then loaded on platinum pans and treated under an oxygen flow of 90 ml min^−1^ while heating to 600 °C at a rate of 10 °C min^−1^. TA Universal Analysis software was used for data analysis.

## Conflicts of interest

There are no conflicts to declare.

## Supplementary Material

RA-008-C8RA00060C-s001

## References

[cit1] Jones F. N. , in Ullmann's Encyclopedia of Industrial Chemistry, 2003

[cit2] Rämänen P., Maunu S. L. (2014). Prog. Org. Coat..

[cit3] Sonnati M. O., Leclair A., Romand A., Choule O., Coggio W. D., Florent N. (2014). Paint Coat. Ind..

[cit4] Won J. Y., Sohn H. J., Song R. H., Woo S. I. (2009). ChemSusChem.

[cit5] de Jesus S. S., Santana A., Ponce G. H. S. F., Maciel Filho R. (2017). J. Chem. Technol. Biotechnol..

[cit6] Mialon L., Pemba A. G., Miller S. A. (2010). Green Chem..

[cit7] Sousa A. F., Vilela C., Fonseca A. C., Matos M., Freire C. S. R., Gruter G. M., Coelho J. F. J., Silvestre A. J. D. (2015). Polym. Chem..

[cit8] Sousa A. F., Fonseca A. C., Serra A. C., Freire C. S. R., Silvestre A. J. D., Coelho J. F. J. (2016). Polym. Chem..

[cit9] Lammens T. M., Franssen M. C. R., Scott E. L., Sanders J. P. M. (2012). Biomass Bioenergy.

[cit10] Ikeda M. , Amino Acid Production Processes in Microbial Production of l-Amino Acids, in Advances in Biochemical Engineering/Biotechnology, ed. R. Faurie, *et al.*, Springer, Berlin, Heidelberg, 2003, vol. 79 10.1007/3-540-45989-8_1 12523387

[cit11] Kumar R., Vikramachakravarthi D., Pal P. (2014). Chem. Eng. Process..

[cit12] Nunes R. S., Cavalheiro É. T. (2007). J. Therm. Anal. Calorim..

[cit13] Dighe A. K., Toliwal S. D., Khotpal R. R. (2000). J. Sci. Ind. Res..

[cit14] Nuplex product information sheets , http://www.nuplex.com/Corporate/coating-resins/technologies/technology?regionId=1&techId=1, accessed July 2017

[cit15] Mare H. E. D. L. (1960). J. Org. Chem..

[cit16] IUPAC , Compendium of Chemical Terminology, “Aldimines”, 2nd edn, 1997

[cit17] Kumarathasan R., Rajkumar A. B., Hunter N. R., Gesser H. D. (1992). Prog. Lipid Res..

[cit18] Bouwman E., van Gorkum R. J. (2007). J. Coat. Technol. Res..

[cit19] Schotten C. (1884). Eur. J. Inorg. Chem..

[cit20] Sato Y., Ikekawa N. (1959). J. Org. Chem..

[cit21] Yu M., Stevenson K., Zhou G. (2014). Tetrahedron Lett..

