# Supplementary material for: Synthesis and characterisation of alkyd resins with glutamic acid-based monomers
Source: RSC Adv. 2018 Feb 21;8(15):8220–7. doi: 10.1039/c8ra00060c (PMC9078555; doi:10.1039/c8ra00060c)
Supplement: RA-008-C8RA00060C-s001 [file RA-008-C8RA00060C-s001.pdf]

Electronic Supplementary Material (ESI) for RSC Advances.
This journal is © The Royal Society of Chemistry 2018

Synthesis and characterization of alkyd resins with glutamic acid-based monomers

Joris Hulsbosch,[a] Laurens Claes,[a] Dries Jonckheere,[a] Dirk Mestach,[b] and Dirk E. De Vos*[a]

Supporting information

1. Characterization of glutamic acid-based monomers

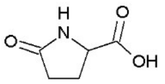

5

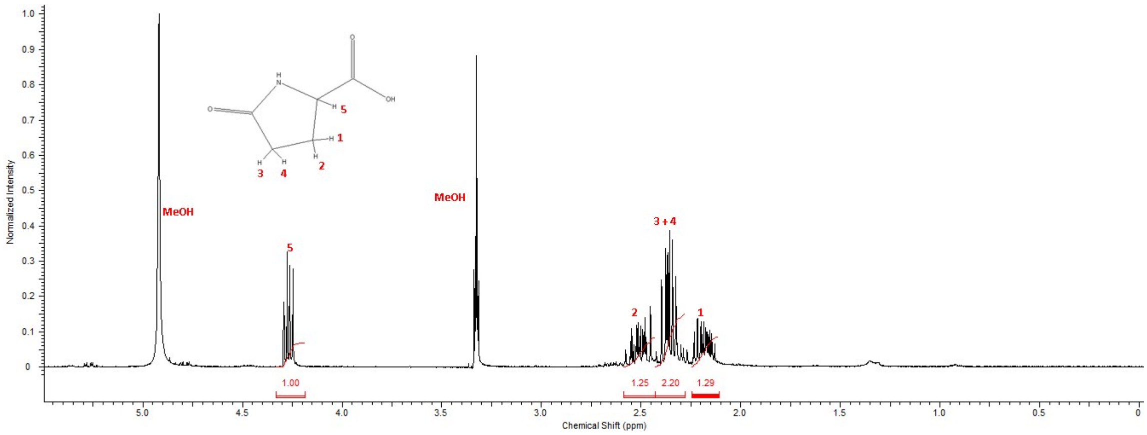

Fig. S1 ^{1}H NMR spectrum of pyroglutamic acid (**5**)(5 mg sample dissolved in 0.7 ml CD_3OD).

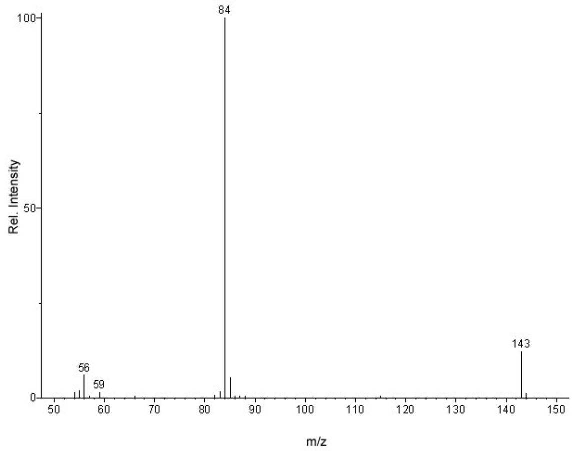

Fig. S2 Mass spectrum of pyroglutamic acid (**5**).

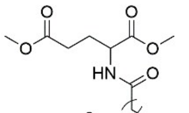

3

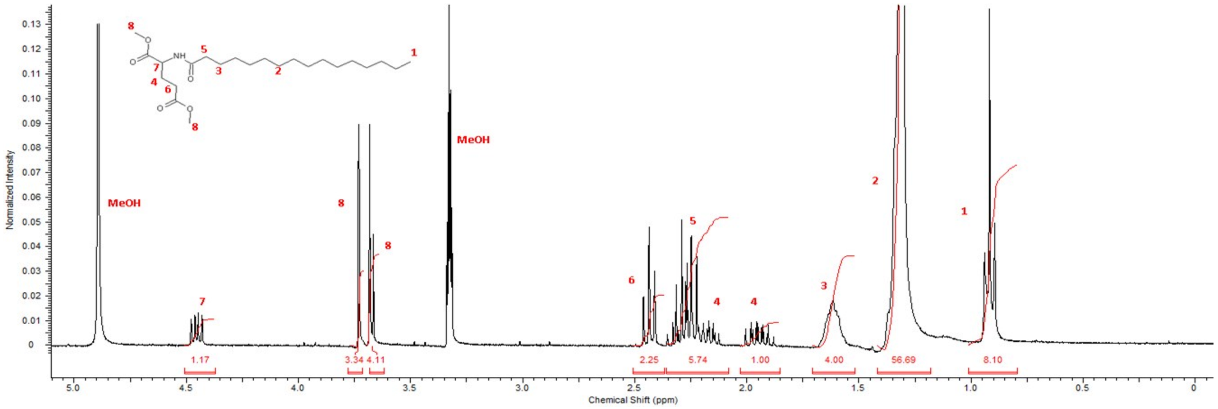

Fig. S3 ^{1}H NMR spectrum of *N*-palmitoylglutamic acid dimethyl ester (**3**; 5 mg sample dissolved in 0.7 ml $CDCl_3$).

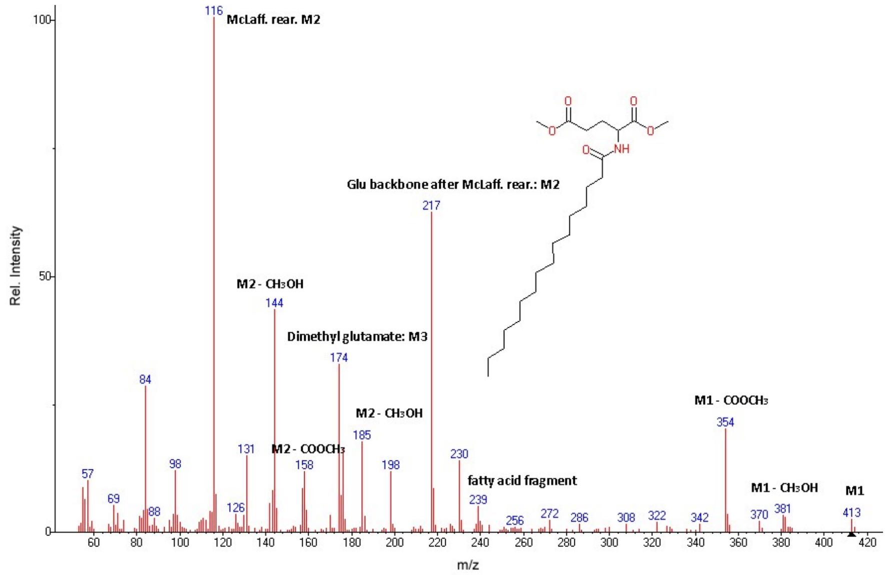

Fig. S4 Mass spectrum of *N*-palmitoylglutamic acid dimethyl ester (**3**).

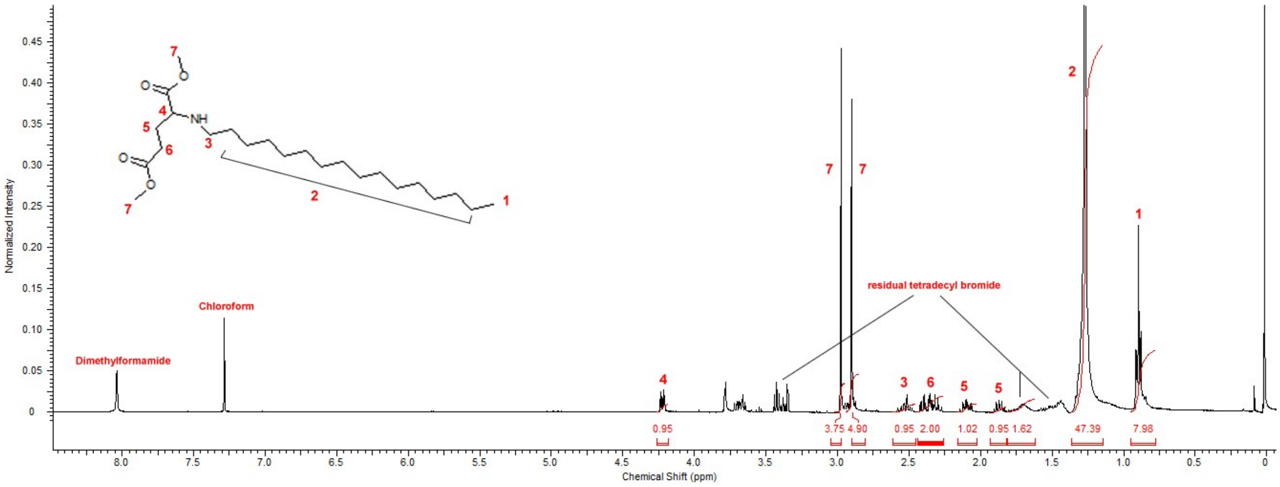

Fig. S5 ^{1}H NMR spectrum of *N*-hexadecylglutamic acid dimethyl ester (**4**; 5 mg sample dissolved in 0.7 ml $CDCl_3$).

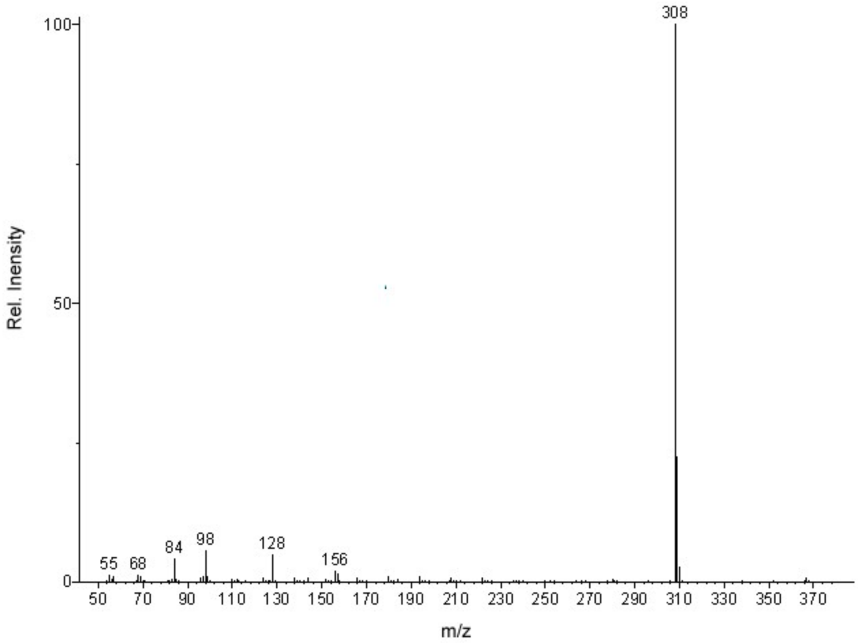

Fig. S6 Mass spectrum of *N*-hexadecylglutamic acid dimethyl ester (**4**).

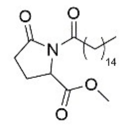

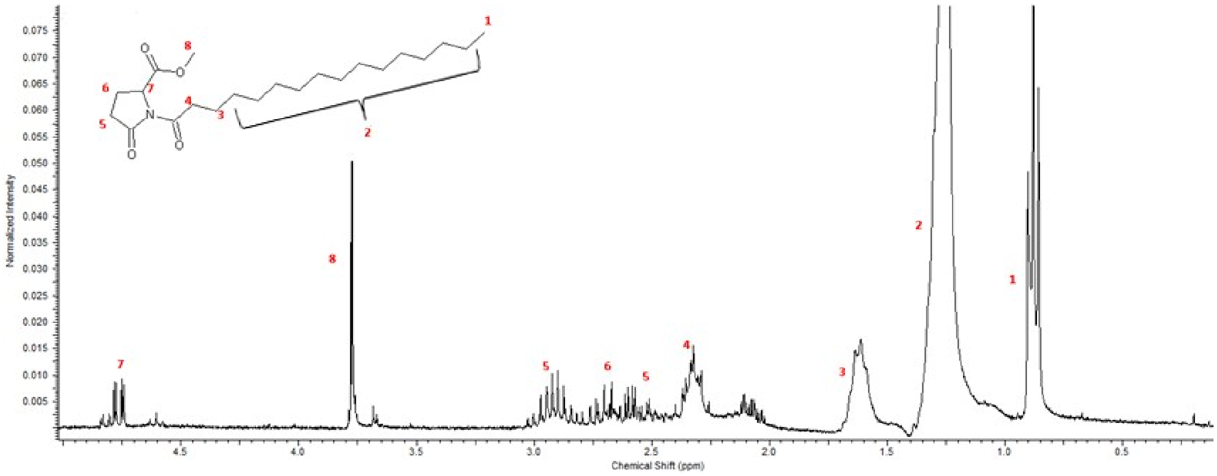

Fig. S7 ^{1}H NMR spectrum of *N*-palmitoylpyroglutamic acid methyl ester (5 mg sample dissolved in 0.7 ml $CDCl_3$).

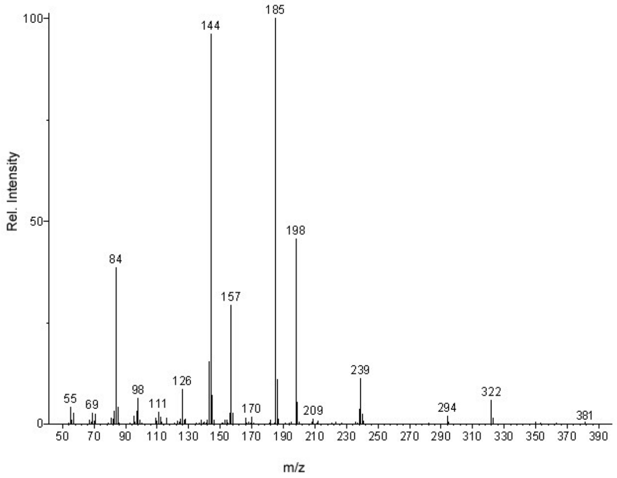

Fig. S8 Mass spectrum of *N*-palmitoylpyroglutamic acid methyl ester.

2. Characterization of alkyd resins

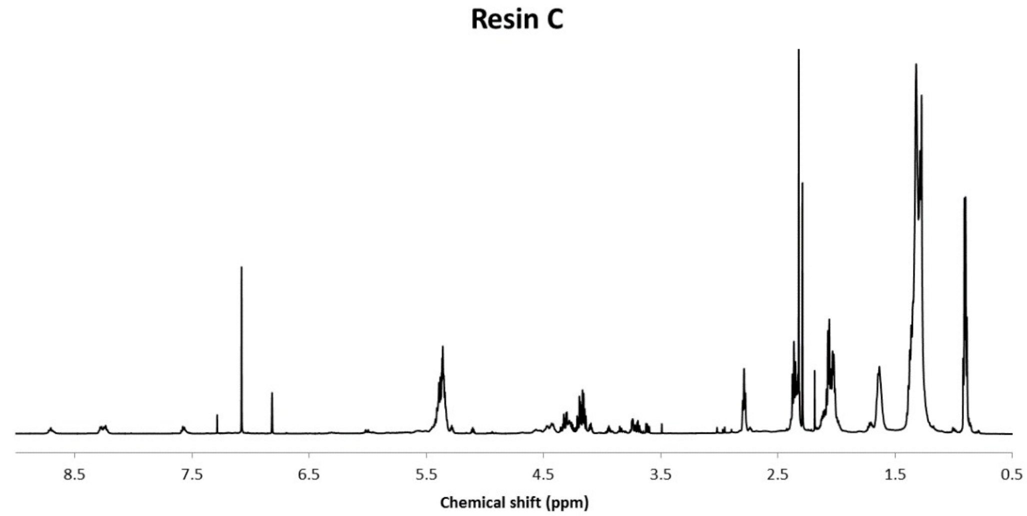

Fig. S9 600 MHz ^{1}H NMR spectrum of resin **C**.

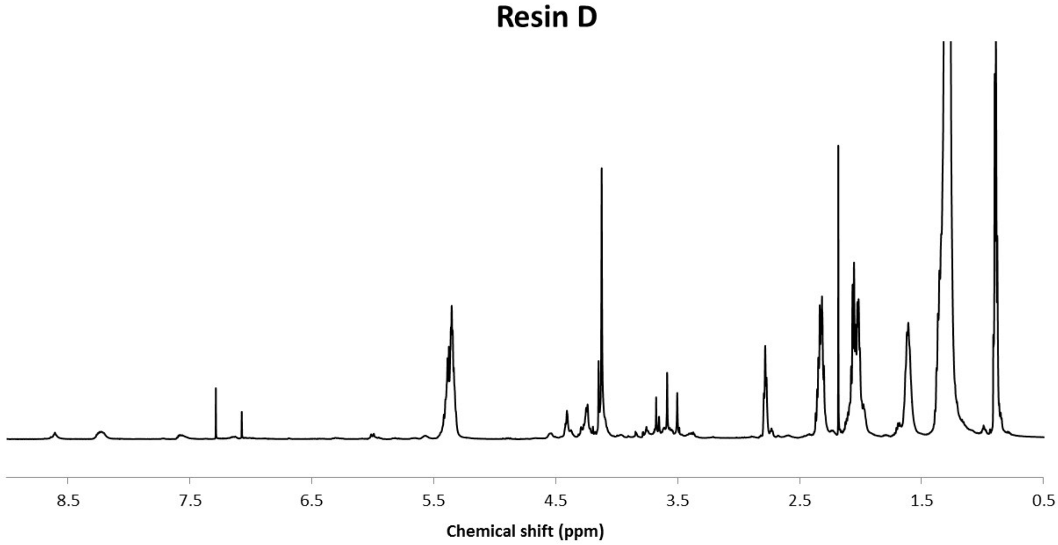

Fig. S10 600 MHz ^{1}H NMR spectrum of resin **D**.

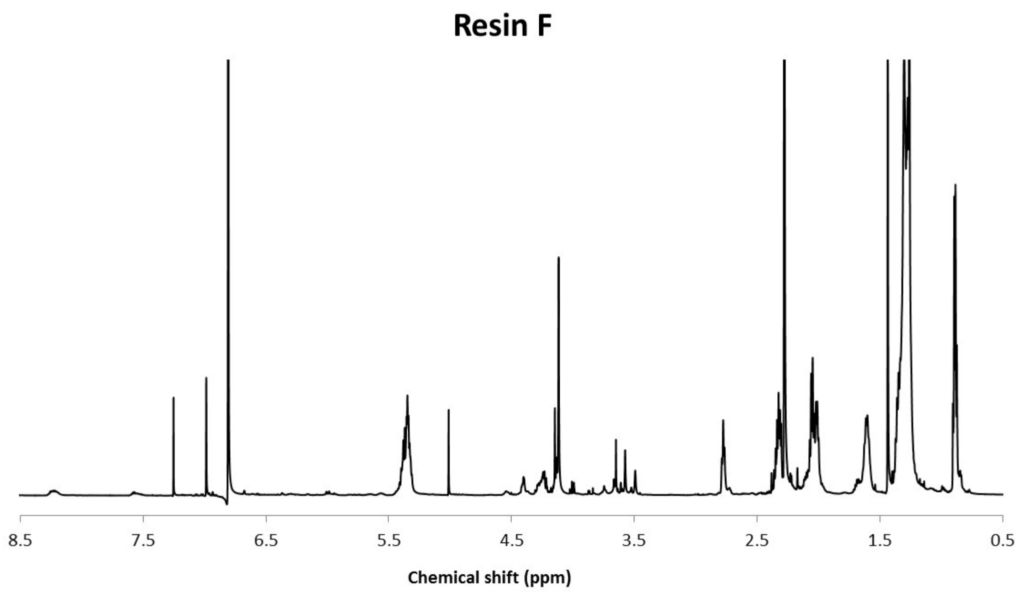

Fig. S11 600 MHz ^{1}H NMR spectrum of resin **F**.

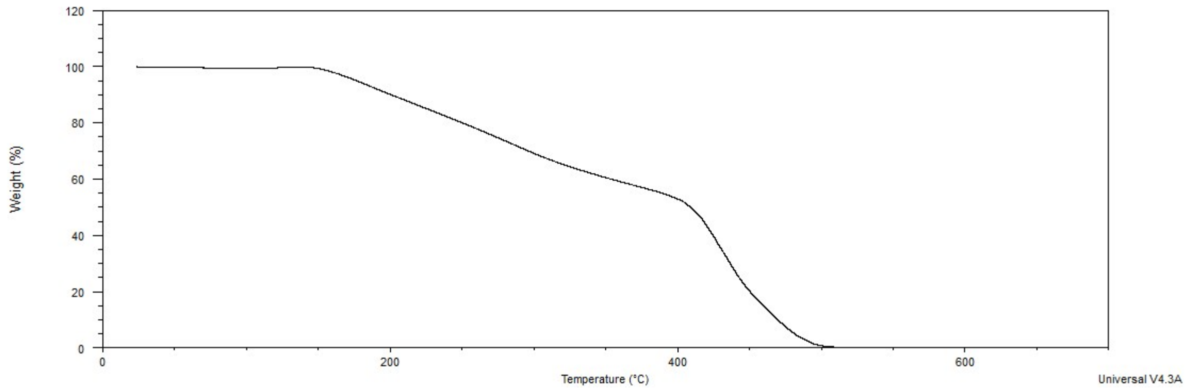

Fig. S12 Thermogravimetric analysis of resin **B**.

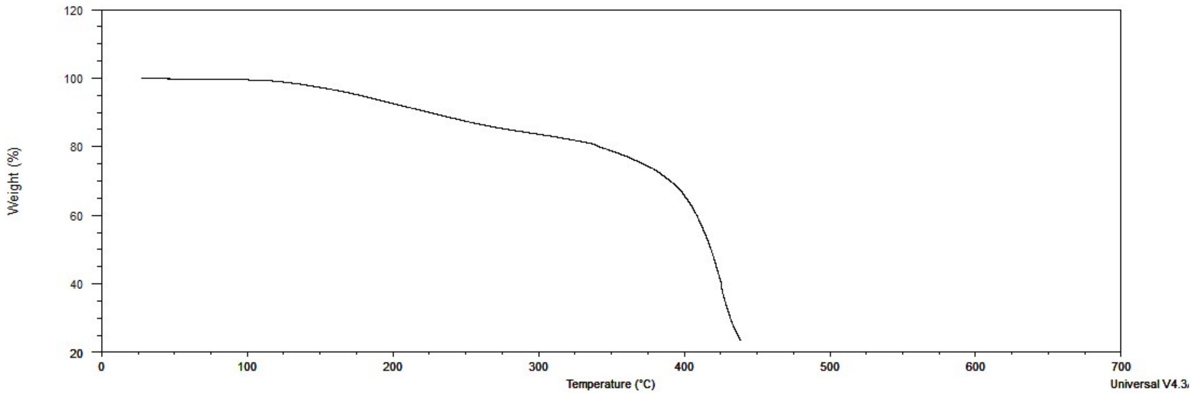

Fig. S13 Thermogravimetric analysis of resin **D**.

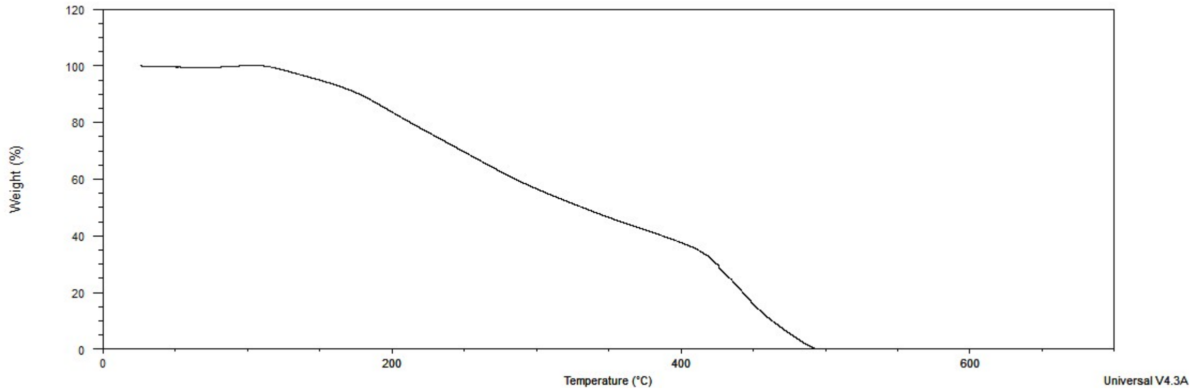

Fig. S14 Thermogravimetric analysis of resin **G**.
